# Immunogenicity of Virus Like Particle Forming Baculoviral DNA Vaccine against Pandemic Influenza H1N1

**DOI:** 10.1371/journal.pone.0154824

**Published:** 2016-05-05

**Authors:** Yong-Dae Gwon, Sehyun Kim, Yeondong Cho, Yoonki Heo, Hansam Cho, Kihoon Park, Hee-Jung Lee, Jiwon Choi, Haryoung Poo, Young Bong Kim

**Affiliations:** 1 Department of Bio-industrial Technologies, Konkuk University, Neungdong-ro, Gwangjin-gu, Seoul, Republic of Korea; 2 Viral Disease Research Center, Korea Research Institute of Bioscience and Biotechnology, Daejeon, Republic of Korea; Shanghai Medical College, Fudan University, CHINA

## Abstract

An outbreak of influenza H1N1 in 2009, representing the first influenza pandemic of the 21st century, was transmitted to over a million individuals and claimed 18,449 lives. The current status in many countries is to prepare influenza vaccine using cell-based or egg-based killed vaccine. However, traditional influenza vaccine platforms have several limitations. To overcome these limitations, many researchers have tried various approaches to develop alternative production platforms. One of the alternative approach, we reported the efficacy of influenza HA vaccination using a baculoviral DNA vaccine (AcHERV-HA). However, the immune response elicited by the AcHERV-HA vaccine, which only targets the HA antigen, was lower than that of the commercial killed vaccine. To overcome the limitations of this previous vaccine, we constructed a human endogenous retrovirus (HERV) envelope-coated, baculovirus-based, virus-like-particle (VLP)–forming DNA vaccine (termed AcHERV-VLP) against pandemic influenza A/California/04/2009 (pH1N1). BALB/c mice immunized with AcHERV-VLP (1×10^7^ FFU AcHERV-VLP, i.m.) and compared with mice immunized with the killed vaccine or mice immunized with AcHERV-HA. As a result, AcHERV-VLP immunization produced a greater humoral immune response and exhibited neutralizing activity with an intrasubgroup H1 strain (PR8), elicited neutralizing antibody production, a high level of interferon-γ secretion in splenocytes, and diminished virus shedding in the lung after challenge with a lethal dose of influenza virus. In conclusion, VLP-forming baculovirus DNA vaccine could be a potential vaccine candidate capable of efficiently delivering DNA to the vaccinee and VLP forming DNA eliciting stronger immunogenicity than egg-based killed vaccines.

## Introduction

Influenza A virus is a notable public health threat that has left its footprints in history [1, 2]. The most recent example, an outbreak of influenza H1N1 in 2009 (sH1N1), represents the first influenza pandemic of the 21^st^ century, designated influenza A/CA/4/2009 (pH1N1) [3, 4]. The first cases were reported in Mexico and the United States in April, and by June, the World Health Organization (WHO) had declared a level 6 pandemic [5]. According to the WHO “Global Alert and Response” in 2010, this pandemic infected more than a million individuals and took 18,449 lives [6]. Despite this toll, it is widely believed that influenza vaccination was important in controlling the spread of pH1N1 [7].

Currently, licensed influenza vaccines are egg-based or cell-based, with the former constituting the majority of the vaccine market [8]. However, traditional egg-based influenza vaccines have several limitations, including vulnerability of supply chain, the necessity for selecting strains a priori, allergic reactions to egg proteins in the vaccine, and an often time-consuming production process—all important issues that need to be addressed [9]. To overcome these limitations, many researchers have tried various approaches to develop alternative production platforms [10–14]. One such alternative approach is baculoviruses, which do not replicate or impose any apparent cytotoxicity in mammalian cells, thus minimizing possible side effects [15, 16].

Baculoviruses have a cloning capacity as large as 38 kb, allowing them to accommodate a single large insert or multiple genes encompassing regulatory elements [17]. These attributes have fueled interest in exploring baculoviruses as vectors for recombinant protein expression systems and gene therapy [18–20]. In spite of the advantages of baculovirus, overcoming their lower efficacy relative to that of conventional vaccines remains a challenge [21]. We previously reported that a non-replicable baculovirus vector containing antigen-encoding DNA could serve as a nano-delivery system, and improve exogenous gene delivery into human cells by incorporating the envelope glycoprotein of human endogenous retrovirus (HERV-W) on recombinant baculovirus [21–23].

Virus-like particles (VLPs) represent an advanced vaccine platform with enhanced immunogenicity [24]. VLPs are formed by structural viral proteins, which have an inherent tendency to self-assemble and mimic the morphology of the pathogen [25]. In contrast to live viruses, VLPs are non-infective and non-replicating, since they are essentially devoid of pahogenetic material [24]. Moreover, VLPs have been known to enhance immunogenicity by presenting antigenic epitopes in correct conformation, resulting in strong humoral and cellular immune responses [24, 26, 27]. Given these advantages, VLPs have been widely used for vaccine development and other biomedical applications [28].

Previously, we confirmed the efficacy of an influenza vaccine developed for DNA delivery of the pH1N1 HA gene using a non-replicable baculoviral vector (AcHERV-HA). However, the AcHERV-HA vaccine only elicited an immune response to HA proteins. Such monovalency may limit the degree of protection against heterologous influenza viruses [21]. Here, we constructed a baculovirus carrying pH1N1 HA, NA, and M1 genes that is able to assemble VLPs in mammalian cells. The efficacy of the VLP-forming baculovirus vaccine—AcHERV-VLP—was tested in BALB/c mice.

## Materials and Methods

### 1. Ethics Statement

This study was performed in strict accordance with the Guide for the Care and Use of Laboratory Animals of the National Institutes of Health. Animal husbandry and experimental procedures were approved by the Konkuk University Institutional Animal Care and Use Committee (IACUC approval No.: KU14082). Splenectomy and pneumonectomy were performed on sterilized dissecting pan. To minimize suffering, mice were sedated by mixture of tiletamine and xylazine anesthesia (50 and 5 mg/kg of body weight, respectively).

### 2. Cells and Viruses

*Spodoptera frugiperda* 9 (Sf9) insect cells (Invitrogen, USA, Carlsbad, CA, Catalog No. 11496–015) were maintained at 27°C in Sf-900 medium (Invitrogen) supplemented with 1% antibiotics/antimycotics (Gibco-BRL, CA, USA).

HEK 293T cells (American Type Culture Collection [ATCC], Manassas, VA, USA, Catalog No. CRL-3216) were cultured in Dulbecco’s modified Eagle’s medium (DMEM; Gibco-BRL) supplemented with 10% fetal bovine serum (FBS; Gibco-BRL) and 1% penicillin-streptomycin. Madin-Darby canine kidney (MDCK) cells (ATCC, Catalog No. PTA-6500) were grown in Eagle’s minimum essential medium (MEM; Gibco-BRL) containing 10% FBS and 1% penicillin and streptomycin. The cells were maintained in a humidified 5% CO_2_ atmosphere at 37°C.

Mouse-adapted influenza virus type A/CA/04/2009 (ma-pH1N1) was kindly provided by the International Vaccine Institute (IVI, Seoul, Republic of Korea). Influenza virus type A/PR/8/1934 (H1N1) was kindly provided by the Centers for Disease Control and Prevention (CDC, Osong, Republic of Korea). The viruses were amplified in 10-day-old embryonated eggs. After incubating at 37°C for 3 days and chilling at 4°C for 12 hours, the allantoic fluid was harvested, aliquoted, and stored at -80°C until use.

### 3. Animals

Female BALB/c mice (17.4±0.9 g), aged 4–5 weeks (n = 48, n refers to number of animals, mouse VAF report indicated that the mice were free of known viral, bacterial and parasitic pathogens) were purchased from Orient-Bio (Seungnam, Kyonggi-do, Republic of Korea) and housed in filter top cages, with water and food provided *ad libitum*.

Mice were maintained in a Bio-safety Level 2 facility with an inverse 12 hours day-night cycle with lights on at 8:30pm in a temperature (22±1°C) and humidity (55±5%) controlled room. All cages contained wood shavings and bedding.

### 4. Construction of Recombinant Baculoviruses

A recombinant baculoviral vector expressing HERV *env* (pFastBac1-HERV) was previously constructed by inserting a synthetic, codon-optimized envelope gene of HERV type W (GenBank accession number NM014590; GenScript, USA) into pFastBac1 (Invitrogen) [29].

The full length of HA and NA protein sequences of influenza A/CA/04/2009 (pH1N1) were previously constructed by polymerase chain reaction (PCR)-amplification from pH1N1 virus cDNA and inserted into the pGEM-T Easy vector (Promega, USA) and were optimized by nucleotide sequencing [21]. The M1 protein sequence of H5N2 influenza A/chicken/Vietnam/OIE-2215/2012 was from NCBI GenBank (accession number BAP71888.1). Genes were codon optimized for optimal expression in mammalian cells and biochemically synthesized (GenScript, USA). Full-length HA, NA, and M1 genes were individually cloned into pcDNA3.1 (-) plasmids under the control of the cytomegalovirus (CMV) promoter (Invitrogen). The HA, NA and M1 genes, from the CMV promoter to the bovine growth hormone (BGH) poly A site, were amplified by PCR and cloned into pFastBac1-HERV plasmid. The primers for PCR are shown in S1 Table.

**Fig 1:**
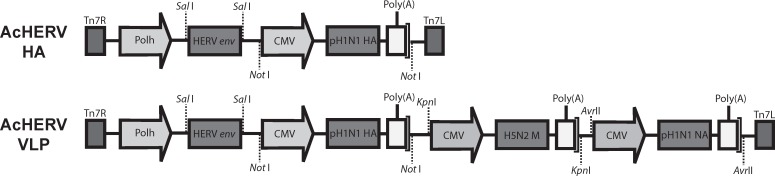
Schematic diagrams of recombinant baculoviruses. Diagram of the AcHERV-HA baculovirus containing HERV *env* and HA genes, the AcHERV-VLP virus additionally including NA and M1 genes under the control of the polyhedron promoter and CMV promoter, respectively. The recombinant baculoviruses were generated using the Bac-to-Bac baculovirus expression system.

Recombinant baculoviruses were produced using the Bac-to-Bac baculovirus expression system according to the manufacturer’s manual (Invitrogen). The scheme for constructing the recombinant baculoviruses, AcHERV-HA and AcHERV-VLP, is shown in Fig 1.

After propagation in Sf9 cells, the amplified recombinant baculoviruses in supernatant were loaded on top of 30% sucrose, and purified by ultracentrifugation at 40,000 rpm at 4°C for 1 hour in a 50.2Ti rotor (Beckman Coulter Inc., Brea, CA, USA). The virus pellet was re-suspended in phosphate-buffered saline (PBS) and stored at -80°C until use.

### 5. Characterization of Baculovirus Expression in Mammalian Cells

The construction of recombinant baculoviral vector vaccines was confirmed by isolating viral genomic DNA from recombinant baculovirus, harvested from infected Sf9 cell using the Nucleospin RNA virus kit (Macherey-Nagel, Düren, Germany), and amplifying HERV, HA, NA, and M1 genes by PCR using primers shown in S1 Table. The quantification of recombinant baculovirus was carried out from recombinant baculovirus DNA using BacPAK™ qPCR Titration Kit (Takara & Clontech, Mountain View, CA, USA), and quantified PCR copy number per milliliter (/ml) was converted to focus forming units (FFU)/ml by according to manufacturer manual.

The expression of baculovirus in mammalian cells was tested by infecting 293T cells with baculovirus at a multiplicity of infection (MOI) of 10. Seventy-two hours after infection, western blotting was carried out using a mouse anti-pH1N1 primary antibody (1:100 dilution; raised in our laboratory from survived mice against ma-pH1N1 challenge). An anti-β-actin antibody (1:2,000 dilution; Santa Cruz Biotechnology, Santa Cruz, CA, USA) was used as a protein loading control.

For immunofluorescence analyses, monolayers of 293T cells were infected with baculovirus at an MOI of 10 for 72 hours. Next, cells were treated with a mouse anti-pH1N1 primary antibody (1:100 dilution) for 2 hours, and incubated with fluorescein isothiocyanate (FITC)-conjugated goat anti-mouse IgG secondary antibody (1:200 dilution; Santa Cruz Biotechnology,). Images of immune-stained cells were obtained using an inverted microscope (Eclipse Ti-U; Nikon, Japan).

### 6. Electron Microscopy

Influenza VLP formation in mammalian cells was verified by harvesting VLPs from growth medium supernatant and lysates of baculovirus-infected 293T cells, and concentrating and partially purifying them using a 20% (w/v) sucrose step gradient in phosphate buffered saline (PBS). Purified VLPs were adsorbed by flotation onto a freshly discharged, 300 mesh formvar/carbon-coated copper grid and allowed to dry completely (Ted pella, Redding, CA, USA). The grids were rinsed with buffer consisting of 20 mM Tris (pH 7.4) and 120 mM KCl, negatively stained with 1% uranyl acetate (Sigma-Aldrich, St. Louis, MO, USA), and then dried by aspiration. VLPs were visualized on a JEM-1010 transmission electron microscope (JEOL Ltd., Tokyo, Japan) operating at 80 kV, and were digitally captured with an ES1000W Erlangshen CCD camera (Gatan Inc., Pleasanton, CA, USA).

### 7. Mouse Immunization and Challenge

Five-week-old female BALB/c mice were divided into four immunization groups (n = 12 mice/group): (1) PBS control (100 μl), (2) killed vaccine (2.0 μg killed vaccine), (3) AcHERV-HA (1×10^7^ FFU/50 μl) and (4) AcHERV-VLP (1×10^7^ FFU/50 μl). All mice received two immunizing doses at 2-weeks intervals by intramuscular injection (days 0 and 14, hind limb), serum samples were collected days 0, 7 and 21 after first immunization (day 0). Serum samples were obtained by centrifugation of whole blood collected from right external jugular vein. Also, two mice per group (n = 2/group) were sedated and sacrificed with efforts to minimize their suffering, then their spleens were removed on days 28.

Two weeks after the final immunization, randomized mice (n = 10/group) were transferred to a biological safety level 2 facility, where they were sedated (mixture of tiletamine and xylazine; 50 and 5 mg/kg of body weight, respectively) and challenged intranasally with ma-pH1N1 at 20-times the 50% lethal dose (20LD_50_) and monitored for 14 consecutive days. Four days after infection, subset of mice (n = 2/group) were sacrificed and their lungs were collected (for analysis of viral shedding) and also stained with hematoxylin and eosin (H&E). These experiments were performed by following designed experiment timelines (S1 Fig).

A 20LD_50_ dose challenge obviously results in severe disease characterized by huddling, ruffled fur, lethargy, anorexia leading to weight loss, and death. Therefore, mice were monitored twice a day. In case of mice showed both typical infection symptoms and rapid weight loss of 15–20 percent within a few days; considered a death. Further, mice showed weight loss over 15–20%, were humanely euthanized using carbon dioxide under condition of mixture of tiletamine and xylazine anesthesia according to the NC3Rs ARRIVE guidelines for the euthanasia of animals.

### 8. Serological Assays

Enzyme-linked immunosorbent assays (ELISAs) were performed to assess anti-pH1N1 specific IgG levels in mouse sera. In brief, blood samples were collected from mice and sera were obtained. Ninety-six well plates were coated overnight with 8 hemagglutination units (HAU) of inactivated pH1N1 using standard methods, as previously described [30, 31]. The absorbance at 450 nm (A450) was measured using an ELISA plate reader (Bio-Rad, Hercules, CA, USA). Results are expressed as reciprocals of the final detectable dilution.

Hemagglutination inhibition (HAI) assays were performed by incubating 4 HAUs of influenza pH1N1 virus with 2-fold diluting heat-inactivated sera in V-bottom 96-well plates and using 1% chicken erythrocytes. The HAI titer is presented as the reciprocal of the highest dilution of serum that completely inhibited hemagglutination.

### 9. Neutralization Assay

The standard micro-neutralization assay was modified from a previously described procedure [30, 32]. Briefly, serum samples were heat-inactivated for 30 minutes at 56°C, and then 2-fold serial dilutions were prepared in a 50 μl volume of serum free MEM (V diluent) in immunoassay plates. The diluted sera were mixed with an equal volume of V diluent containing influenza virus at 100-times the median tissue culture infective dose (TCID_50_). After 2-hour incubation, the mixture was transferred to duplicate wells of MDCK cells grown in 96-well tissue culture plates. After virus control wells exhibited an advanced virus-induced cytopathic effect (CPE; 48 hours), the neutralizing capacity of individual serum samples was assessed by determining the presence or absence of CPE, and staining with 1% crystal violet solution (1% formaldehyde and 10% methanol) for 15 minutes. Neutralizing antibody titers were expressed as the reciprocal of the highest dilution of serum that completely inhibited virus-induced CPE.

### 10. ELISPOT Assays

The production of interferon-γ (IFN-γ) from the splenocytes of immunized mice was detected by ELISPOT assay kit (BD Biosciences, USA, San Jose, CA), as described by the manufacturer. Briefly, 96-membrane plates were coated with 0.2 μg of mouse IFN-γ capture antibody and blocked with 10% FBS at 37°C. Splenocytes (1 × 10^6^ cells) in 100 μl of RPMI-1640 medium were applied to each well and stimulated with inactivated influenza pH1N1 virus for an additional 24 hours at 37°C. Plates were then washed and treated with 20 ng of biotinylated mouse IFN-γ detection antibody for 2 hours. Streptavidin-alkaline phosphatase was then added to the wells, and color was developed using an AEC substrate reagent (BD Biosciences). The number of spots was counted using an ELISPOT reader (AID ElispotReader ver.4; AID GmbH, Straßberg, Germany).

### 11. Titration of Virus in the Lungs of Challenged Mice

Four days after challenge, mice (n = 2/group) were sacrified and their lung tissue was collected in 3 ml PBS containing 2% gentamycin. Collected lungs were homogenized for approximately 2 minutes using a hand-held tissue homogenizer (Biospec Products, Bartlesville, OK, USA) and centrifuged to remove debris. The supernatant was serially 10-fold diluted and transferred to MDCK cell monolayers in 96-well tissue culture plates and incubated for 2 days at 37°C. The virus titer was calculated using the Reed-Muench formula and was expressed as log10 TCID_50_ per milliliter.

### 12. Statistical Analysis

All statistical analyses were performed using GraphPad 6.0 software (GraphPad Software, Inc. La Jolla, CA, USA), and data were presented as means ± standard error of mean (SEM) or as a percentage. For the analysis of the significance of differences, we used one-way analysis of variance (ANOVA) or two-tailed Student’s *t*-test. *P* values equal to or less than 0.05 were considered statistically significant.

## Results

### Expression of Recombinant Baculoviruses *In Vitro*

**Fig 2:**
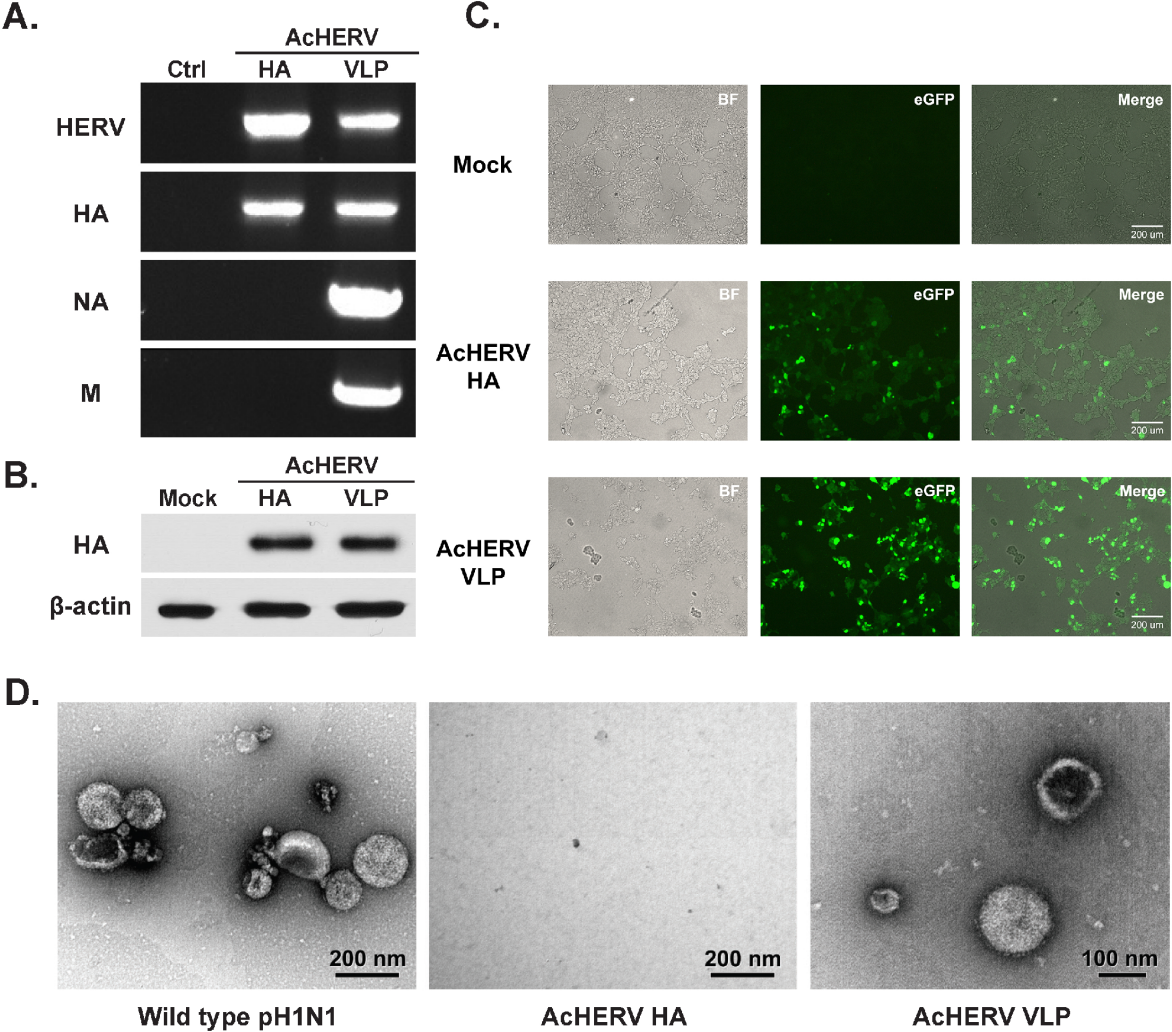
Characterization of the recombinant baculoviruses (rBV; AcHERV-HA and AcHERV-VLP) and their expression in mammalian cells. (A) PCR detection of HERV, HA, NA, and M1 genes in constructed baculovirus DNA. Lane 1: control for PCR; lane 2: AcHERV-HA baculovirus DNA; lane 3: AcHERV-VLP baculovirus DNA. (B) Western blot analysis of HA protein expression in recombinant baculovirus (rBV)-infected 293T cells. Mock, AcMNPV-infected cells; AcHERV-HA, AcHERV-HA–infected cells; AcHERV-VLP, AcHERV-VLP–infected cells. (C) Immunofluorescence micrograph of rBV-infected 293T cells. Seventy-two hours after infection, the cells were incubated with a mouse antibody against pH1N1, followed by incubation with a FITC-conjugated goat anti-mouse IgG antibody. Mock, AcMNPV-infected cells; AcHERV-HA, AcHERV-HA–infected cells; AcHERV-VLP, AcHERV-VLP–infected cells; Merge, merged image. (D) Transmission electron microscopy of negatively stained, purified VLPs from rBV-infected 293T cells. Seventy-two hours after infection, VLPs were harvested from 293T cells, concentrated, and partially purified using 20% step sucrose gradient ultracentrifugation. For electron microscopy, VLPs were stained with 1% uranyl acetate. Scale bar, 100 nm (× 100,000) or 200 nm (× 120,000). Wild-type pH1N1 purified A/California/04/2009 influenza virus; AcHERV-HA, purified VLP from AcHERV-HA–infected 293T cells; AcHERV-VLP, purified VLP from AcHERV-VLP–infected 293T cells.

To efficiently express HA, NA and M1 from baculovirus in mammalian cells, we constructed a HERV-encoded baculovirus expressing HA, NA, and M1 genes under the control of the 5’- CMV promoter in bacmid DNA (Fig 1). Correct insertion of each gene in recombinant baculoviruses was confirmed by PCR amplification from baculoviral genomic DNA (Fig 2A). Sf9 cell-derived baculoviruses containing HA gene or VLP forming genes (AcHERV-HA or AcHERV-VLP), were transduced into 293T cells. HA protein expression in virus-infected 293T cells was detected by western blotting (Fig 2B). Protein expression in baculovirus-infected 293T cells was further confirmed by immunofluorescence staining using a mouse polyclonal antibody against pH1N1 and a fluorescein isothiocyanate (FITC)-conjugated anti-mouse antibody (Fig 2C). Collectively, these results demonstrate that proteins were successfully expressed in 293T cells infected with baculoviruses. Influenza VLP formation in mammalian cells was confirmed by comparing negatively stained AcHERV-VLPs with wild-type pH1N1 (positive control) using transmission electron microscopy, which revealed largely spherical enveloped particles that were consistent with the 100–150 nm diameter (Fig 2D).

### Humoral Immune Responses in Mice

**Fig 3:**
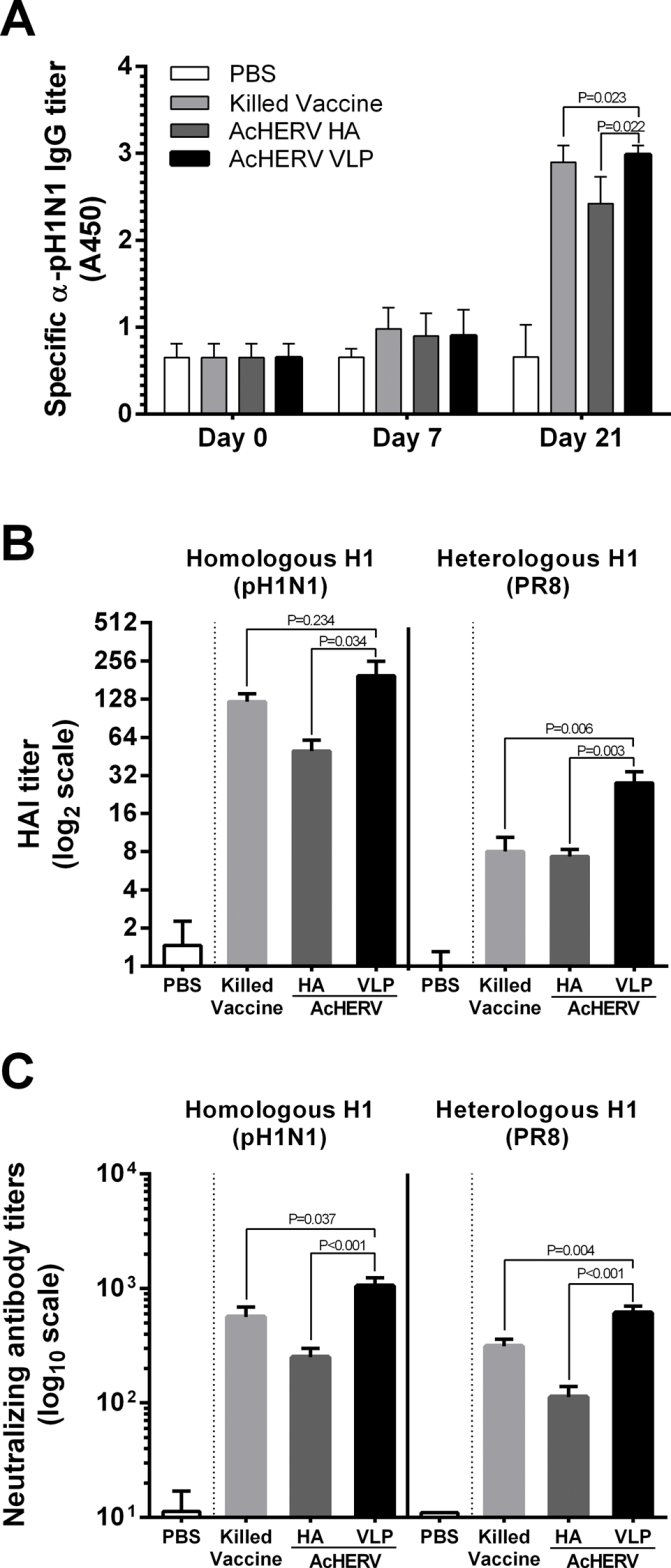
Serological analysis in mice immunized with killed vaccine and baculoviruses. Sera from mice injected intramuscularly with PBS, killed vaccine, AcHERV-HA, or AcHERV-VLP were collected and evaluated for immune response. (A) Antigen-specific IgG antibody titers against pH1N1 in mouse sera (on days 0, 7 and 21) were determined by ELISA. A450 refers absorbance at 450 nm. (B) HAI titer against the pH1N1 strain or PR8 strain in mouse sera on day 21. (C) Neutralizing titer against the pH1N1 strain or PR8 strain in mouse sera on day 21. ELISA and HAI assays were performed using ten randomly selected samples from each group (10/12). Neutralizing assays were performed using eight randomly selected samples from each group (8/12). Values in parentheses indicate number of mice tested/total number of mice immunized in each group. All experiments were run in triplicate. The data shown are means ± SEM for samples. Statistical analysis showed that data were significant with *p*<0.05 or not significant (one way ANOVA and two-tailed Student’s *t*-test): killed vaccine and AcHERV HA groups were compared with AcHERV VLP group (on days 21).

To investigate the immunogenicity of AcHERV-VLP, we collected immunized mouse sera (10/12, ten randomly selected samples from each group) and analyzed the titer of pH1N1-specific IgG by ELISA. As shown in Fig 3A, pH1N1-specific IgG was produced in all vaccination groups after the first immunization and was boosted ~2.5–3-fold after the second immunization. A comparative analysis showed that IgG titer was 1.2-fold higher in AcHERV-VLP–immunized mice than in AcHERV-HA–immunized mice, and was comparable to that in mice immunized with killed vaccine.

To test the cross-reactivity of sera from immunized mice, we performed HAI assays using a homologous H1 strain (pH1N1) and an intrasubgroup H1 strain (PR8). All vaccine immunization groups (except PBS controls) showed HAI antibody titers > 1:32 against the homologous pH1N1; as expected, immunization with AcHERV-VLP enhanced HAI antibody responses (~3.9-fold) compared with immunization with AcHERV-HA (Fig 3B). Interestingly, only AcHERV-VLP–immunized mice showed high HAI responses (>1:32) against intrasubgroup H1 strain (Fig 3B).

To further confirm these results, we determined the neutralizing titer of immunized mouse sera (8/12, eight randomly selected samples from each group) against pH1N1 and PR8. Similar to HAI responses, neutralizing titers in sera was about 4-fold and 5-fold higher in mice immunized with AcHERV-VLP than in mice immunized with AcHERV-HA against pH1N1 virus and PR8 virus, respectively (Fig 3C).

These data indicate that AcHERV-VLP immunization elicits a strong humoral immune response and shows that the resulting antibodies have greater neutralizing potential against other H1 strain.

### IFN-γ Immune Responses in Mice

**Fig 4:**
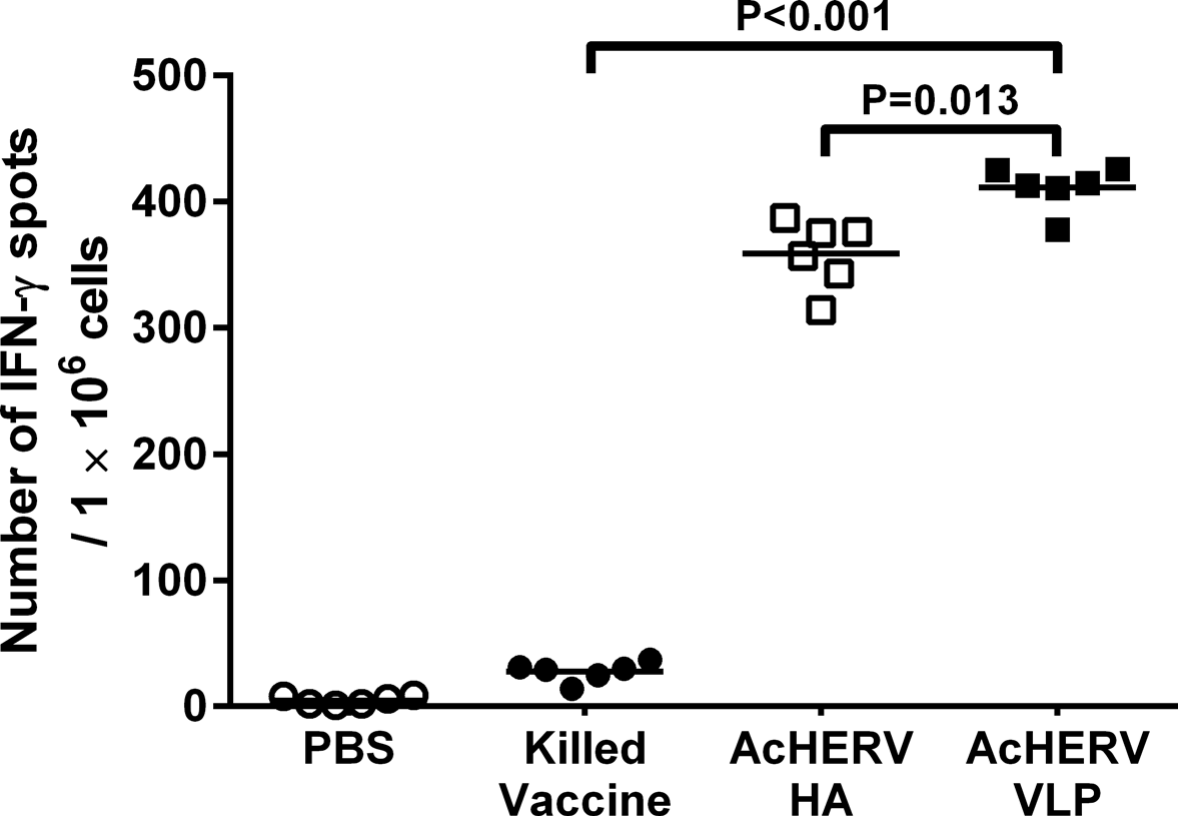
IFN-γ production in mice immunized with killed vaccine and baculoviruses. Two weeks after the final immunization, splenocytes from mice injected intramuscularly with PBS, killed vaccine, AcHERV-HA, or AcHERV-VLP were collected and evaluated for the number of IFN-γ secreted spots from pH1N1-specific T cells in splenocytes. ELISPOT assays were performed using two randomly selected samples from each group (2/12). Values in parentheses indicate number of mice tested/total number of mice immunized in each group. All experiments were run in triplicate. The data shown are actual number of IFN-γ spot as a scatter dot and mean value as a line. Statistical analysis showed that data were significant with *p*<0.05 (ANOVA and two-tailed Student’s *t*-test): killed vaccine and AcHERV HA groups were compared with AcHERV VLP group.

To determine the effect of immunization on IFN-γ production, we performed ELISPOT assays on splenocytes isolated from mice (n = 2/group) 2 weeks after the final immunization. Splenocytes from mice immunized with killed vaccine secreted only basal levels of IFN-γ, similar to results obtained in mice injected with PBS (Fig 4). However, splenocytes from mice immunized with baculoviruses (AcHERV-HA or AcHERV-VLP) secreted 10- to 11-fold more IFN-γ than those from mice immunized with killed vaccine. The comparison between AcHERV-HA group and AcHERV-VLP group showed statistically difference (p = 0.013), and IFN-γ spot number in AcHERV-HA group was 12.8% lower than number in AcHERV-VLP group, which indicates AcHERV-VLP immunization elicits more IFN-γ production than AcHERV-HA immunization. These results support the conclusion that the HERV-encoded, VLP-forming baculovirus system is a strong candidate influenza vaccine with the potential to increase the cellular immune response as well as the humoral immune response.

### Protection against pH1N1 Viral Challenge in Mice

**Fig 5:**
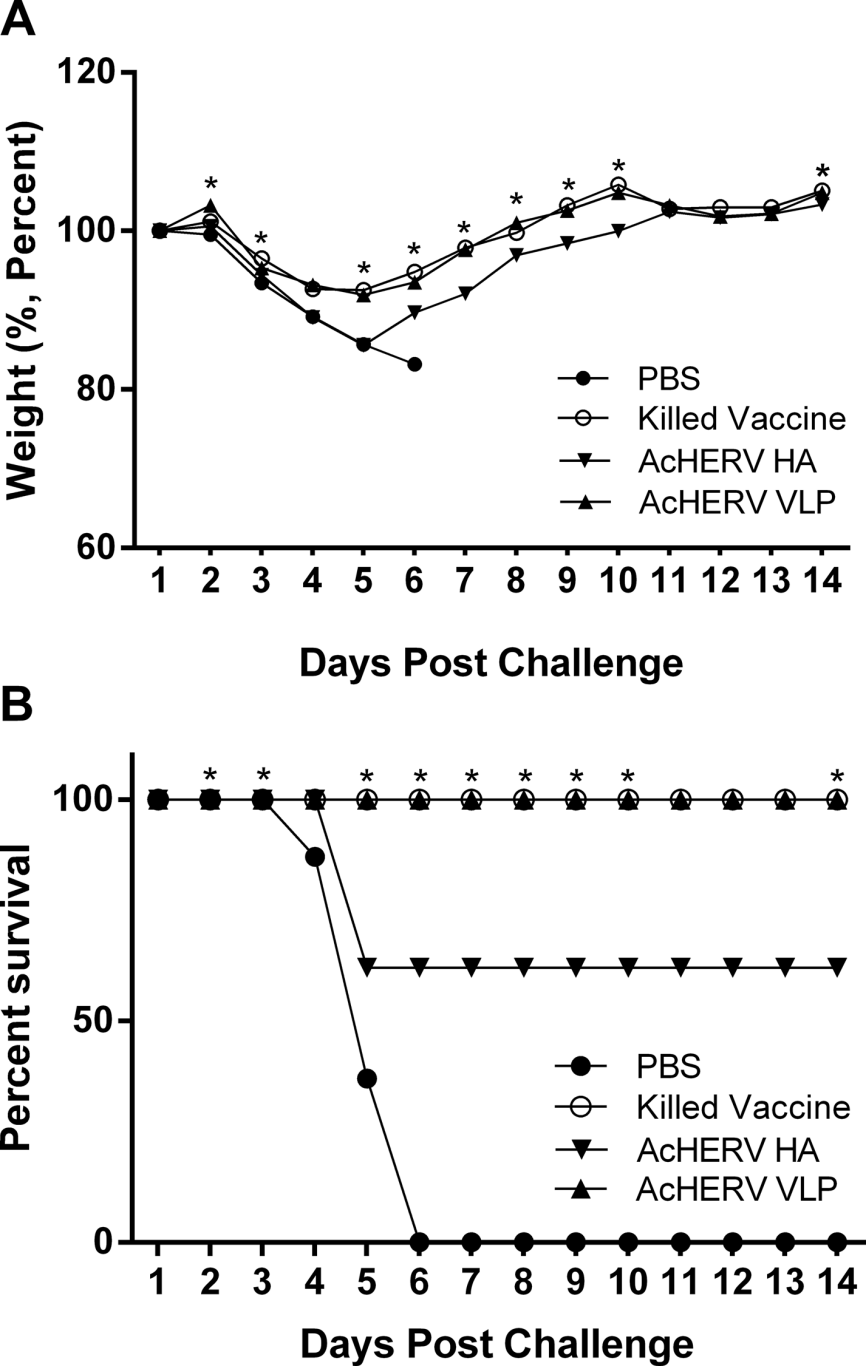
Protective effect of immunization against challenge with a lethal dose of ma-pH1N1. Body weights of mice intranasally challenged with a 20LD_50_ dose of ma-pH1N1 2 weeks after the final immunization were monitored for 14 consecutive days. (A) Percent body weight change after challenge with a 20LD_50_ dose of ma-pH1N1. Changes in body weight (n = 10 mice/group) are expressed as the mean value for each group. (B) Survival rate after challenge with a 20LD_50_ dose of ma-pH1N1. Statistical analysis performed between the AcHERV-HA group and AcHERV-VLP group. Statistical analysis showed that data were significant with *p < 0.05 (two-tailed Student’s *t*-test).

Mice (n = 10/group) were intranasally challenged with a 20LD_50_ dose of infectious pH1N1 virus 3 weeks after immunization, and weight loss and health conditions were monitored for 14 consecutive days. All challenged mice lost weight rapidly during the initial 5 days post infection (dpi), with the exception of mice in the PBS group, subsequently regained lost weight. Mice immunized with AcHERV-VLP lost ~10% of their initial weight, whereas mice in the AcHERV-HA group showed about a 15% weight loss. Furthermore, mice were monitored the entering of recovery phage, AcHERV-VLP began recovery on 7 dpi, whereas recovery was delayed (11 dpi) in the AcHERV-HA group (Fig 5A).

AcHERV-VLP immunized groups showed complete protection against live influenza virus challenge, while AcHERV-HA group showed 62.5% protection; as expected no protection was observed in the PBS group. Collectively, these results indicate that AcHERV-VLP provides better protection than AcHERV-HA, due to enhanced immunogenicity in both humoral immune response and cellular immune response (Fig 5B).

### Histological Analysis of Lungs from Immunized Mice after Viral Challenge

To assess the relationship between viral clearance and histological lesions in the lung, we sacrificed a subset of mice from each group (n = 2) on day 4 post challenge, and then determined the titer of shed virus in the lung and assessed lung damage. Virus titer was determined by quantifying infection of MDCK cells, expressed as log_10_ TCID_50_/ml. Virus titer in PBS-injected mice was 10^4.73 ± 0.22^, whereas that in mice immunized with killed vaccine or AcHERV-HA was 10^3.99 ± 0.23^ and 10^4.08± 0.11^, respectively—approximately a 5-fold reduction in viral titer. Strikingly, the titer of shed viruses in mouse lungs in the group immunized with AcHERV-VLP was even lower (10^3.47 ± 0.21^)—an overall reduction in viral titer close to 20 fold (Table 1).

**Table 1:** Differences in clearance of lung viral particles in vaccinated mice 4 days post-challenge with live ma-pH1N1.

Immunization group	Lung virus titers^a^ (Log_10_ TCID_50_/ml tissue)
PBS	4.73 ± 0.22 (2/2)
Killed vaccine	3.99 ± 0.23 (2/2)
AcHERV-HA	4.08 ± 0.11 (2/2)
AcHERV-VLP	3.47 ± 0.21 (2/2)

^a^Mice were intranasally challenged with 20LD_50_ of ma-pH1N1 virus 14 days after the last immunization. Lung tissue was harvested 4 days post challenge, and viral titers in lung homogenates were determined as described in Section 2.11.

Data are presented as means ± SD of titers of samples. The number of mice that shed virus is indicated in parentheses (number of mice shedding virus/number of mice tested).

**Fig 6:**
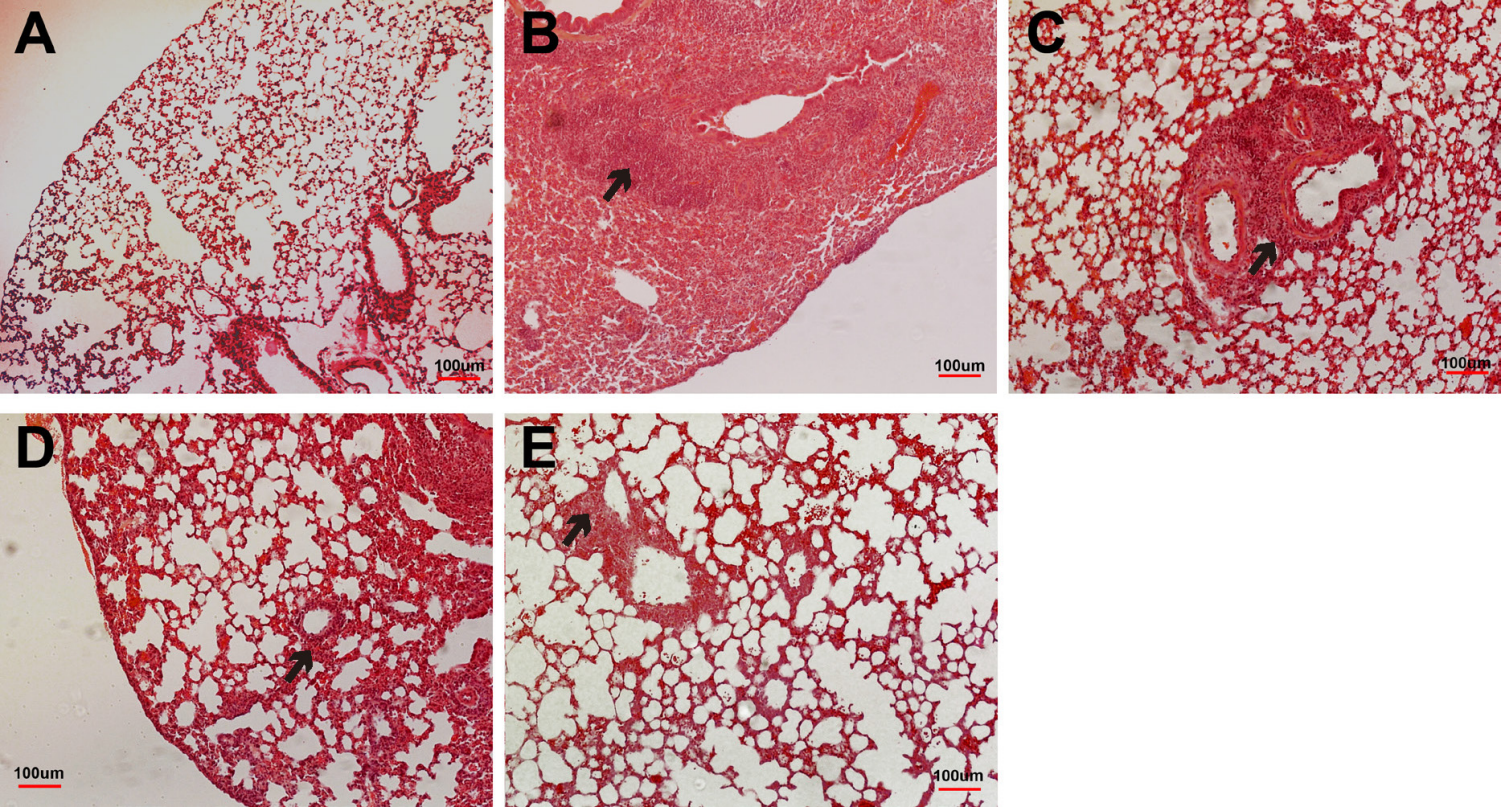
Histological lesions in lung sections from immunized mice after challenge with pH1N1. A subset of mice (n = 2 mice/group) from each group was sacrificed 4 days post challenge, and their lungs were H&E-stained for histological evaluation. (A) Non-infected BABL/c mice; (B) mice injected with PBS; (C) mice vaccinated with killed vaccine; (D) mice vaccinated with AcHERV-HA; (E) mice vaccinated with AcHERV-VLP. Arrows indicate the infiltration of inflammatory cells, including infiltration in vessels, the pulmonary parenchyma, and alveolar septa. Scale bar, 100 μm.

Consistent with viral titer results, histological analyses of H&E-stained lung sections revealed that mice injected with PBS alone had severe infiltration in the vessels, bronchioles and alveoli; even mice immunized with killed vaccine or AcHERV-HA showed interstitial/alveolar infiltration and structural damage around bronchioles or vessels (Fig 6C and 6D). However, mice immunized with AcHERV-VLP had less interstitial and alveolar infiltration, suggesting protection of these structures against viral infection (Fig 6E).

## Discussion

Recent trends in influenza vaccine development have followed two general paths: a universal vaccine approach using the stalk domain to induce a broad-spectrum neutralizing antibody, and a specific approach using VLPs [33, 34]. In addition to their advantages of improved safety and strong immune response, VLP vaccines can readily substitute HA or NA genes to allow rapid, dynamic responses to a new influenza outbreak [14, 35, 36]. However, efficient construction and production of VLPs are major obstacles to commercialization [37].

Previously, we reported on the efficacy of influenza HA vaccination using a HERV encoated-baculoviral DNA vaccine (AcHERV-HA) and showed that its immunogenicity and gene delivery were excellent [21]. Nonetheless, the fact of a vaccine that delivers only the HA gene could not be expected to elicit broad-spectrum neutralizing antibodies has been questioned.

Matrix protein (M) has been researched as an essential VLP forming component, especially M1 part has been reported that induces broad range of cross-reactivity and can incorporate different subtype HA protein or NA protein, resulting in mixed influenza VLPs, and can provide protection against influenza viruses of different subtypes [36, 38, 39]. Therefore, we here investigated mixed VLP forming combination of M1 gene from H5 strain (A/chicken/Vietnam/OIE-2215/2012) with full length of HA and NA genes from H1 strain (A/CA/04/2009) and constructed a VLP-forming, gene-delivering, HERV encoated-baculovirus (AcHERV-VLP) that essentially kills two birds with one stone.

After, AcHERV-VLP transduction in mammalian cells (HEK 293T cells), VLP was purified and confirmed by transmission electron microscope (Fig 2D). This result suggested that our constructed baculovirus was capable of delivering VLP forming DNA to HEK 293T cell and VLP was formed directly inside the cell. Thus, AcHERV-VLP has additional advantage in production process; due to there is no extra purification of VLPs.

Following immunogenicity and efficacy studies in mice, serological analysis showed that, AcHERV-VLP immunization elicited stronger immunogenicity than AcHERV-HA in humoral immune response (Fig 3). Also, among sera obtained from variously immunized mice, only sera from AcHERV-VLP–immunized mice exhibited cross-reactivity against the H1N1 PR8 strain, which has 81.6%, 61.5% and 96.4% homology with the pH1N1 HA, NA and M1 protein, respectively (Fig 3B and 3C). Generally, VLP has an identical structure as a virus, and functions as a mimic antigen [24, 26]. Hence, the advantage of VLP vaccine has been described in conformational way; especially it presents more optimal neutralizing epitopes than that of HA only. Also, AcHERV-VLP includes M1 protein as a VLP component and recent publication indicates that M1 protein contains highly conserved epitopes of influenza A virus inducing cross-reactivity [37, 40]. Thus, AcHERV-VLP induced enhanced antibody response for both of homologous H1 and heterologous H1, whereas AcHERV-HA could not.

Notably no cross-reactivity was observed against H3N2, H5N1, or B type influenza virus (data not shown). The lack of correlation between H5 M1 gene mixed VLP and broad cross-reactivity might suggests that combination of M1 gene from H5 strain with full length of HA and NA genes from H1 strain was not well-matched to induce broad-spectrum humoral immune response in our baculovirus DNA delivery system [39, 41]. Therefore, we sought the key to the enhanced breadth of the neutralizing spectrum of AcHERV-VLP is the inclusion of pivotal immunogenic genes. For example, it has been shown that addition of the extracellular domain of matrix protein 2 (M2e) and several other HA genes can increase the breadth of neutralizing antibodies [42–45].

IFN-γ screening in immunized mice showed that, AcHERV-VLP immunization induced stronger IFN-γ related cellular immune response in splenocyte than AcHERV-HA immunization, which indicates that VLP-forming baculovirus presented more T cell epitopes from NA and M1 proteins, and those epitopes triggered stronger cellular responses than that of HA only by stimulating with whole killed influenza virus (Fig 4.) [38]. Th1 stimulating VLP forming DNA vaccine contains major T cell epitopes in VLP forming components. Not only HA but also influenza NA and M1 protein have conserved CD8+ T cell epitopes [46, 47]. Therefore, VLP forming AcHERV-VLP presented more T cell epitopes to cellular immune cells than HA protein targeted AcHERV-HA.

Further evidence for AcHERV-VLP as an effective vaccine is provided by the observation that challenged mice recovered more rapidly from infection and showed diminished viral shedding in the lung (Fig 5, Fig 6 and Table 1). These findings provide support for the idea that immunization with AcHERV-VLP shows better protection against pandemic influenza virus H1N1 by enhancing the neutralizing antibody response and inducing an enhanced IFN-γ secretion [21].

Additionally, the baculoviral vector containing VLP-forming influenza structural genes has several additional advantages, including ease of manipulation, lack of toxicity, and cost effective production, requiring no addition protein purification or other components. In conclusion, our findings indicate that the VLP-forming AcHERV-VLP vaccine could be considered a new candidate DNA vaccine for use against new emerging influenza virus epidemics

## Supporting Information

S1 Fig Experimental timelines. BALB/c mice were divided into four immunization groups (n = 12 mice/group): (1) PBS control (100 μl), (2) killed vaccine (2.0 μg killed vaccine), (3) AcHERV-HA (1×10^7^ FFU/50 μl) and (4) AcHERV-VLP (1×10^7^ FFU/50 μl) and given i.m. injection (↑). On days 0, 7, or 21 blood collection, splenectomy and pneumonectomy were performed, respectively (↓). Two weeks after immunization, mice were transferred to a biological safety level 2 facility, where they were sedated and challenged intranasally with mouse-adapted influenza virus A/CA/04/2009 (ma-pH1N1) at a 20LD_50_ dose (▲). Mice were observed health condition and weighed for 14 consecutive days.
(TIFF)

S1 File NC3Rs ARRIVE Guidelines checklist. (PDF)

S1 Table Primers used in this study. (A) Primers used for amplification of genes from the pcDNA3.1 vector. (B) Primers used for baculovirus DNA confirmation.
(DOC)
