# Supplementary figures and images for: Immunogenicity of Virus Like Particle Forming Baculoviral DNA Vaccine against Pandemic Influenza H1N1
Source: PLoS One. 2016 May 5;11(5):e0154824. doi: 10.1371/journal.pone.0154824 (PMC4858234; doi:10.1371/journal.pone.0154824)

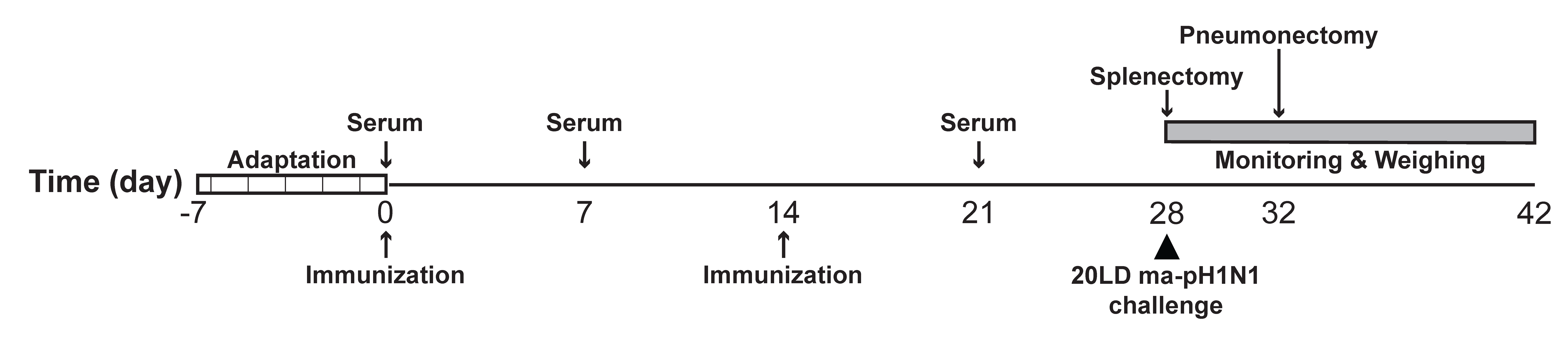

Supplement: S1 Fig — BALB/c mice were divided into four immunization groups (n = 12 mice/group): (1) PBS control (100 μl), (2) killed vaccine (2.0 μg killed vaccine), (3) AcHERV-HA (1×10^7^ FFU/50 μl) and (4) AcHERV-VLP (1×10^7^ FFU/50 μl) and given i.m. injection (↑). On days 0, 7, or 21 blood collection, splenectomy and pneumonectomy were performed, respectively (↓). Two weeks after immunization, mice were transferred to a biological safety level 2 facility, where they were sedated and challenged intranasally with mouse-adapted influenza virus A/CA/04/2009 (ma-pH1N1) at a 20LD50 dose (▲). Mice were observed health condition and weighed for 14 consecutive days. (TIFF) [file pone.0154824.s001.tiff]
