# Supplementary material for: Immunogenicity of Virus Like Particle Forming Baculoviral DNA Vaccine against Pandemic Influenza H1N1
Source: PLoS One. 2016 May 5;11(5):e0154824. doi: 10.1371/journal.pone.0154824 (PMC4858234; doi:10.1371/journal.pone.0154824)
Supplement: S1 Table — (A) Primers used for amplification of genes from the pcDNA3.1 vector. (B) Primers used for baculovirus DNA confirmation. (DOC) [file pone.0154824.s003.doc]

**S1 Table. Primers used in this study**

|  | **Primer name** | **Sequence (5’ to 3’)** |
| --- | --- | --- |
| A | F-NotI-HA | AAA**GCGGCCG**CTGTACGGGCCAGATATAC |
|  | R-NotI-HA | AAA**gcggccg**cagaagccatagagcccac |
|  | F-AvrII-NA | AAA**CCTAGG**TGTACGGGCCAGATATAC |
|  | R-AvrII-NA | AAA**CCTAGG**AGAAGCCATAGAGCCCAC |
|  | F-KpnI-M | AAA**GGTACC**TGTACGGGCCAGATATAC |
|  | R-KpnI-M | AAA**GGTACC**AGAAGCCATAGAGCCCAC |
| B | F-HERV | TTTGTTCGCCCAGGACTCTA |
|  | R-HERV | GTTTACCCCGCGCCACCTTCTCTAGGCA |
|  | F-HA | CACCGATGAAGGCAATACTAGTAGTTCTGC |
|  | R-HA | TTAAATACATATTCTACACTGTAGAGACCC |
|  | F-NA | CACCGATGAATCCAAACCAAAAGATAATAACCATT |
|  | R-NA | TTACTTGTCAATGGTAAATGGCAACTCAGC |
|  | F-M | AAGTCGAAACCTATGTCCTGAGCA |
|  | R-M | GATAAGCCTGCAGGTTTTCCAGCA |

(A) Primers used for amplification of genes from the pcDNA3.1 vector. (B) Primers used for baculovirus DNA confirmation.

Note: underlined and bolded letters indicate introduced restriction enzyme sites for cloning of PCR product.
